# Supplementary material for: Comprehensive Characterization of fucAO Operon Activation in Escherichia coli
Source: Int J Mol Sci. 2024 Apr 2;25(7):3946. doi: 10.3390/ijms25073946 (PMC11011485; doi:10.3390/ijms25073946)
Supplement: Supplementary file 1 [file ijms-25-03946-s001.zip › Table_S1_Strain_PfucAO.pdf]

Supplementary Table S1. Strains and plasmids used in this study

Strains or plasmids	Genotype or description	Reference
**Strains**		
BW25113	*E. coli* K12 strain, wild type	[56]
ZZ200	Δ*lacI*, Δ*lacZ* and Δ*lacY* in BW25113	This study
ZZ201	Δ*fucR* in ZZ201	This study
ZZ202	Δ*srsR* in ZZ201	This study
Δ*crp* Glp$^+$	Δ*crp* in BW25113, able to grow on glycerol	[57]
ZZ203	Δ*crp* in ZZ200, Glp$^+$	This study
ZZ204	*fucAO* operon *lacZ* reporter, Cm^R	This study
ZZ205	Δ*crp* in ZZ204, Glp$^+$	This study
ZZ206	Δ*fucR* in ZZ204	This study
ZZ207	Δ*srsR* in ZZ204	This study
ZZ208	P_{fucAO} driving *lacZ* at the *fuc* position in ZZ204	This study
ZZ209	P_{fucO} driving *lacZ* at the *fuc* position in ZZ204	This study
ZZ210	P_{fucAO}.hc driving *lacZ* at the *fuc* position in ZZ204	This study
ZZ211	P_{fucAO} (-546 to +30) driving *lacZ* at the *lac* position	This study
ZZ212	P_{AO}.V2 (-480 to +30) driving *lacZ* at the *lac* position	This study
ZZ213	P_{AO}.V3 (-377 to +30) driving *lacZ* at the *lac* position	This study
ZZ214	P_{AO}.V4 (-339 to +30) driving *lacZ* at the *lac* position	This study
ZZ215	P_{AO}.V5 (-270 to +30) driving *lacZ* at the *lac* position	This study
ZZ216	P_{AO}.V6 (-206 to +30) driving *lacZ* at the *lac* position	This study
ZZ217	P_{AO}.V7 (-166 to +30) driving *lacZ* at the *lac* position	This study
ZZ218	P_{AO}.V8 (-123 to +30) driving *lacZ* at the *lac* position	This study
ZZ219	P_{fucO} driving *lacZ* at the *lac* position	This study
ZZ220	P_{fucAO}.hc driving *lacZ* at the *lac* position	This study
ZZ221	P_{AO}.V5 mutated in O_{Crp}2 driving *lacZ* at the *lac* position	This study
ZZ222	P_{AO}.V5 mutated in O_{Crp}3 driving *lacZ* at the *lac* position	This study
ZZ223	P_{AO}.V5 mutated in O_{FucR}2 driving *lacZ* at the *lac* position	This study
ZZ223	P_{AO}.V5 mutated in O_{FucR}3 driving *lacZ* at the *lac* position	This study

**Plasmids**		
pZA31	IS5P*bgl-bglG* cloned downstream of the *rrnB*T in pKDT	[58]
pKDT	A *rrnB* terminator (*rrnB*T) in pKD13	[59]
pKDT_P_{fucAO}	P_{fucAO} (-546 to +30 relative to the *fucA* start site) cloned into pKDT	This study
pKDT_P_{AO}.V2	P_{AO}.V3 (-486 to +30 relative to the *fucA* start site) cloned into pKDT	This study
pKDT_P_{AO}.V3	P_{AO}.V3 (-377 to +30 relative to the *fucA* start site) cloned into pKDT	This study
pKDT_P_{AO}.V4	P_{AO}.V4 (-339 to +30 relative to the *fucA* start site) cloned into pKDT	This study
pKDT_P_{AO}.V5	P_{AO}.V5 (-270 to +30 relative to the *fucA* start site) cloned into pKDT	This study
pKDT_P_{AO}.V6	P_{AO}.V6 (-206 to +30 relative to the *fucA* start site) cloned into pKDT	This study
pKDT_P_{AO}.V7	P_{AO}.V7 (-166 to +30 relative to the *fucA* start site) cloned into pKDT	This study
pKDT_P_{AO}.V8	P_{AO}.V8 (-123 to +30 relative to the *fucA* start site) cloned into pKDT	This study
pKDT_P_{fucO}	P_{fucO} (-449 to +30 relative to the *fucO* start site) cloned into pKDT	This study
pKDT_P_{fucAO}.hc	P_{fucAO} (-546 to -147 relative to the *fucA* start site) cloned into pKDT	This study
pKDT_P_{AO}.ΔO_{Crp}2	P_{AO}.V5 with ΔO_{Crp}2 cloned into pKDT	This study
pKDT_P_{AO}.ΔO_{Crp}3	P_{AO}.V5 with ΔO_{Crp}3 cloned into pKDT	This study
pKDT_P_{AO}.ΔO_{FucR}2	P_{AO}.V5 with ΔO_{FucR}2 cloned into pKDT	This study
pKDT_P_{AO}.ΔO_{FucR}3	P_{AO}.V5 with ΔO_{FucR}3 cloned into pKDT	This study
