# Supplementary material for: Comprehensive Characterization of fucAO Operon Activation in Escherichia coli
Source: Int J Mol Sci. 2024 Apr 2;25(7):3946. doi: 10.3390/ijms25073946 (PMC11011485; doi:10.3390/ijms25073946)
Supplement: Supplementary file 1 [file ijms-25-03946-s001.zip › Table_S2_Oligos_PfucAO.pdf]

Supplementary Table S2. Oligonucleotides used in this study

Name	Sequence	Use
lacI1-P1	ggcatgcatttacgttgacaccatcgaatggcgcaaaacctttcgcggtatgtgtaggctggagctgcttc	Deletion of *lacI, lacZ* and *lacY*
lacY2-P2	ctgacgacgcagcagggaaagcgggccggggccgctaagcgtgaacacggaaattccggggatccgtcgacctg	Deletion of *lacI, lacZ* and *lacY*
fucR1-P1	agcggcacgccagcaagcgatagtcgacctgctgctgaaccataccagcctgtgtaggctggagctgcttc	Deletion of *fucR*
fucR2-P2	gaagttgcaacctggcgctcatcagaaataatgtgcgttacctcatccagattccggggatccgtcgacctg	Deletion of *fucR*
srsR1-P1	ggatggacatttttatttcgaaaaagatgcgcaatttcattttattagcgtgtgtaggctggagctgcttcg	Deletion of *srsR*
srsR2-P2	gggttaatgactcgtcccgtcacgatcttcagcaatatgctgaagcatcgcatatgaatatcctccttagttc	Deletion of *srsR*
Fuc.lacZ-F	gaagcaacgcttgaggatattgtagagctttaccataccgcctggtaaatgcgcacaggaaacagctatgaccatgattacg	Amplification of *lacZ* for constructing ZZ204
cat.lacZ-R	caacgtctcattttcgccagattatttttgacaccagaccaactggtaatggtag	Amplification of *lacZ* for constructing ZZ204
Z.cat-F	ctaccattaccagttggtctggtgtcaaaaataatctggcgaaaatgagacgttgatc	Amplification of *cat* for constructing ZZ204
Fuc.Cat-R	gcatatcgacggcaatttacagcaattgcggttggtacggtaacggcgagcttacgccccgccctgccactcatcgcag	Amplification of *cat* or "*lacZ:cat*" for constructing ZZ204, ZZ208 and ZZ210
PfucA-Z-Fn	gaatcagagagaggtagctaatggaacgaaataaacttgctcgtcagatttaacacaggaaacagctatgaccatgattacgggctgcttc	Amplification of "*lacZ:cat*" cassette for constructing ZZ208
PAO-rrnT-F	ggtaattaaacggctaattcaatagtgtgaaaggaacaacattattgccctgtgtaggctggagctgcttc	Insertion of the *rrnB* terminator T1 between P_{fucAO} and *fucA* in ZZ204 to make ZZ209
PAO-rrnT-R	aatctgacgagcaagtttatttcgttccattagctacctctctctgattcaaaggttcatcgcgctcgagacgca	Insertion of the *rrnB* terminator T1 between P_{fucAO} and *fucA* in ZZ204 to make ZZ209
PAO.hc-Z-F	gcggtcatttttaatggggttataaaaagagaacttgcagttatttactgtcacaggaaacagctatgaccatgattacgggctgcttc	Amplification of "*lacZ:cat*" cassette for constructing ZZ210
GSP-fucA-R	tttccaggcaagtgtcaataatctgacgag	*fucA* specific primer for cDNA amplification
PfucAO-Xho-F	atactcgagagcatcctcttaggttcagaagcttaag	Cloning of P_{fucAO} (-546 to +30) into pKDT
PfucAO-Bam-R	ttaggatccttaaatctgacgagcaagtttatttcgttcc	Cloning of P_{fucAO} and its 7 shorter versions into pKDT
PAO.V2-Xho-F	atactcgagttcgggcttcatatcagtgaatatttg	Cloning of P_{fucAO} (-480 to +30) into pKDT
PAO.V3-Xho-F	ttactcgagtagctagaaggtgactttatgtgactac	Cloning of P_{fucAO} (-377 to +30) into pKDT
PAO.V4-Xho-F	attctcgagataatcgaaatttcatgcccgtttcag	Cloning of P_{fucAO} (-339 to +30) into pKDT
PAO.V5-Xho-F	aatctcgagtattaattgtttgtgccgaaaacggtc	Cloning of P_{fucAO} (-270 to +30) into pKDT
PAO.V6-Xho-F	atactcgagaaatatttttaagagcggtcatttttaatg	Cloning of P_{fucAO} (-206 to +30) into pKDT
PAO.V7-Xho-F	tatctcgagaacttgcagttatttactgtgattag	Cloning of P_{fucAO} (-166 to +30) into pKDT
PAO.V8-Xho-F	aatctcgagatataagcttgtgaatttctttcttg	Cloning of P_{fucAO} (-123 to +30) into pKDT
PfucO-Xho-F	tccatatggcagcctatcaaagcag	Cloning of P_{fucO} into pKDT

PfucO-Bam-R	cgtttcgttcagaatcattctgttagc	Cloning of P_{fucO} into pKDT
PfucA1-Z-P1	gcatttacgttgacaccatcgaatggcgcaaaacctttcgcggtatgtgtaggctggagctgcttc	Amplification of "*km*[r]:T:P_{fucAO}" cassette for construction of P_{fucAO} driving *lacZ* at the *lac* loucus
PfucA2-Z-P2	gtaaaacgacggccagtgaatccgtaatcatggtcatagctgtttcctgtgttaaatctgacgagcaagtttatttc gttcc	Amplification of "*km*[r]:T:P_{fucAO}" cassette for construction of P_{fucAO} driving *lacZ* at the *lac* loucus
OCrp2-R	ttcacaagcttatatttcagcgcctggtagaactaatcacagtaaataactg	Mutation of $O_{Crp}2$ in P_{AO}.V5
OCrp2-F	actgtgattagttctaccaggcgctgaaatataagcttgtgaatttc	Mutation of $O_{Crp}2$ in P_{AO}.V5
OCrp3-R	aacagggcaataaatctgttccttgaggcctattgaattagccgtttaattac	Mutation of $O_{Crp}3$ in P_{AO}.V5
OCrp3-F	aacggctaattcaataggcctcaaggaacagatttattgccctgttttgaatc	Mutation of $O_{Crp}3$ in P_{AO}.V5
OFucR2-R	ctattcctccaagcccggaagcaaacgtttcggcacaaacatgagattggtg	Mutation of $O_{FucR}2$ in PAO.V5
OFucR2-F	caccaatctcatgtttgtgccgaaacgtttgcttccgggcttggaggaatagaa	Mutation of $O_{FucR}2$ in PAO.V5
OFucR3-R	ctctttttataaccccattaaaaagcgaacctcttaaaaatatttatcaaaacgg	Mutation of $O_{FucR}3$ in PAO.V5
OFucR3-F	ccgttttgataaatatttttaagaggttcgctttttaatggggttataaaaagag	Mutation of $O_{FucR}3$ in PAO.V5
